# Comparative effects of three different physical education teaching modes on college students' physical fitness during the COVID-19 pandemic: A longitudinal study

**DOI:** 10.1016/j.heliyon.2024.e28305

**Published:** 2024-03-27

**Authors:** Zhixuan Tao, Ergang Zhu, Xugui Sun, Jun Sun

**Affiliations:** Department of Public Foundation, Wannan Medical College, 241000, Wuhu, China

**Keywords:** Classroom teaching, Online teaching, Blended teaching, Physical education, Physical fitness

## Abstract

**Background:**

An appropriate teaching mode in physical education is crucial for ensuring effective education outcomes. Given the dynamic nature of the COVID-19 pandemic, teaching modes are often adjusted. However, there is a lack of in-depth research on the impact of different teaching modes on the outcomes of physical education. Our study aims to address this gap by conducting a comparative analysis of the teaching effectiveness of three different physical education modes among Chinese college students, with a focus on evaluating their impact on physical fitness.

**Method:**

This study adopted a longitudinal retrospective observational design. We systematically examined the three stages of the COVID-19 pandemic (stage 1: September 2020 to January 2021; stage 2: September 2021 to January 2022 and stage 3: February 2022 to July 2022), along with the three corresponding physical education teaching modes (classroom teaching, online teaching and blended teaching) and administered three physical fitness tests (T1, T2 and T3). The physical fitness test included 7 indicators: body mass index, vital capacity, 50-m run, standing long jump, sit-and-reach, pull-ups (male), 1000-m run (male), sit-ups (female) and 800-m run (female). A mixed ANOVA model was used to analyse the physical fitness test indicators across the three different teaching modes.

**Results:**

A total of 3363 college students (1616 males and 1747 females) enrolled in 2020 completed the three physical fitness tests. Most students were aged between 17 and 20 years old, and the BMI criteria indicated a normal distribution. The results indicated that there were significant differences in the overall training effectiveness for all students across vital capacity (p < 0.001, η^2^ = 0.077), sit-and-reach (p < 0.001, η^2^ = 0.027), and middle and long-distance running (p < 0.001, η^2^ = 0.031). Post-hoc multiple comparison analyses further revealed that the blended teaching was the most effective in improving these fitness indicators, whereas the online teaching performed poorly on the training effects of middle and long-distance running. Significant training effects were also shown for sit-ups (p < 0.001, η^2^ = 0.192) for females and pull-ups (p < 0.001, η^2^ = 0.020) for males in gender-specific physical fitness indicators. Similarly, blended teaching showed superior results to other teaching modes.

**Conclusion:**

These findings emphasize the importance of conducting online physical education during unforeseen public health events and highlight the comprehensive effects of blended physical education in the post-pandemic era. Future initiatives should prioritize targeted interventions to address the observed variations in various physical fitness indicators under different physical education teaching models.

## 1 Introduction

The sudden outbreak of the COVID-19 pandemic has caused the largest disruption in education in history [1]. By mid-July 2020, universities in more than 160 countries were closed, and more than 1 billion students were affected [2]. Classroom teaching worldwide has been forced to stop, and prompting educational institutions to adopt online courses in response to the pandemic [3,4]. At the end of May 2021, more than 1454 universities in China launched online courses, offering approximately 1.07 million courses to a total of 17.75 million college students [5,6].

However, the large number of online courses has led to an increase in students' sedentary time [7]. Simultaneously, quarantine measures implemented due to the pandemic have resulted in a decrease in physical activity [8]. These factors collectively contribute to a decline in students' physical fitness [7,8]. This trend transcends mere changes in learning and lifestyle, extending to broader health considerations. Extended periods of sedentary behavior elevate the risk of various health issues, including cardiovascular and musculoskeletal diseases [9,10]. Furthermore, a reduction in physical activity may impact students' mental health, potentially leading to an increase in academic stress levels [11,12].

The observed decline in physical fitness underscores the necessity for proactive intervention in physical education by schools. Due to the unpredictability of the COVID-19 pandemic, schools worldwide have adopted three teaching modes: classroom teaching, online teaching, and blended teaching [13]. Classroom teaching is characterized by direct, face-to-face interactions between educators and students [14]. It offers significant advantages in terms of visualization, immediate response, and in-person discussions [15]. Online teaching refers to teaching activities conducted by teachers and students through the Internet. The biggest advantage of online teaching is that it overcomes the limitations of time and space [16]. The blended teaching mode refers to the combination of classroom teaching and online teaching. The critical factor in ensuring the effectiveness of blended teaching is striking a balance between classroom teaching and online learning [17].

Comparative study on the effects of teaching modes shows that different physical education teaching modes may result in different health outcomes. Studies show that classroom teaching was irreplaceable, as it allows teachers to comprehensively observe students' reactions during the learning process, thereby achieving educational objectives through adjusting teaching methods and content [18,19]. Moreover, in the context of physical education classes, students can fully experience the sports atmosphere and boost their enthusiasm for physical activities [20]. However, classroom teaching requires teachers to transfer knowledge to students in a limited timeframe, so it is not always efficient for students who have different abilities to learn knowledge in a limited timeframe [21]. Unlike classroom teaching, online teaching can provide students with video resources for repeated learning [16]. Online teaching has potential as a low-cost and scalable home program and as well as a potential means of maintaining physical activity during self-isolation [22]. According to the latest report from the American College of Sports Medicine, online training courses during the pandemic in 2021 emerged as the predominant trend in global physical fitness exercises, it integrates social support mechanisms and provide techniques for acquiring behavioral skills, both of which are crucial components of health behavior change theories [23]. Studies show that online teaching plays a significant role in enhancing the physical fitness of obese children and improving the physical and mental health of sedentary adults during the COVID-19 pandemic [24,25]. However, it is differentially attractive to people according to physical activity levels, BMI, age, and cultural [26]. Moreover, the lack of face-to-face guidance, supervision, and timely feedback may result in less effective learning outcomes for students in online teaching [27,28].

The unprecedented challenges brought about by the COVID-19 pandemic have forced educational institutions to adopt alternative teaching modes. This shift has prompted questions about the effectiveness of these changes in the context of physical education, highlighting the need for a thorough review of the implemented modes to assess their efficacy. However, few studies have focused on the impact of different teaching modes on physical education outcomes during the COVID-19 pandemic. The aim of this study was to provide insight into the effect of different teaching modes on college students' education through comparative analysis of physical fitness test indicators. Another purpose is to provide a decision-making basis for future physical education teaching. Therefore, we propose the following hypothesis: H_1_: There is no significant difference in the impact of different teaching modes on each indicator of physical fitness. H_2_: There is no significant difference in the physical fitness test scores of male and female students under the same teaching mode. H_3_: There is no interaction effect between teaching mode and gender on the indicators of physical fitness.

## 2 Methods

### 2.1 Subjects

A total of 3363 students at Wannan Medical College who were enrolled in 2020 were included in the study. The study population included 1616 males and 1747 females. The basic demographic characteristics of the participants under the different teaching modes are shown in Table 1. This study was approved by the Research Ethics Review Committee of Wannan Medical College (Reference Number: 2022-093). Informed consent was obtained from all the participating students.

**Table 1:** The basic demographic characteristics of participants at three times.

Variables	Male(n = 1616)			Female(n = 1747)		
	T1	T2	T3	T1	T2	T3
	Mean (SD)	Mean (SD)	Mean (SD)	Mean (SD)	Mean (SD)	Mean (SD)
Age(years)	18.4(0.9)	19.4(0.9)	19.9(0.9)	18.3(0.8)	19.3(0.8)	19.8(0.8)
Height(cm)	176.0(5.8)	176.5(5.7)	176.6(5.5)	164.0(5.3)	164.1(5.2)	164.2(5.1)
Weight(kg)	70.2(13.2)	69.7(12.7)	69.9(12.5)	56.2(8.7)	55.7(8.6)	55.4(8.0)

### 2.2 Design

**Fig. 1:**
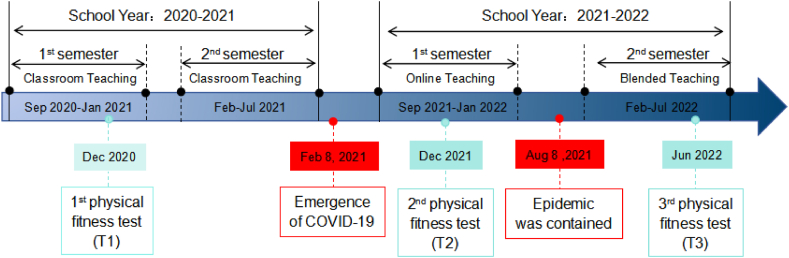
The summary of epidemic events, teaching modes and physical fitness test times.

This was a longitudinal retrospective observational study of the 2020 cohort. We reviewed the three stages of the COVID-19 pandemic (stage 1: September 2020 to January 2021; stage 2: September 2021 to January 2022; stage 3: February 2022 to July 2022), along with the corresponding physical education teaching modes (classroom teaching, online teaching, blended teaching) and three physical fitness tests (T1, T2, T3). Fig. 1 shows the three stages of the COVID-19 pandemic and the corresponding teaching modes and physical fitness tests. We analysed the impact of the three teaching modes on college students' physical fitness by comparing the results of physical fitness tests under the different teaching modes.

#### 2.2.1 Setting and participants

**Fig. 2:**
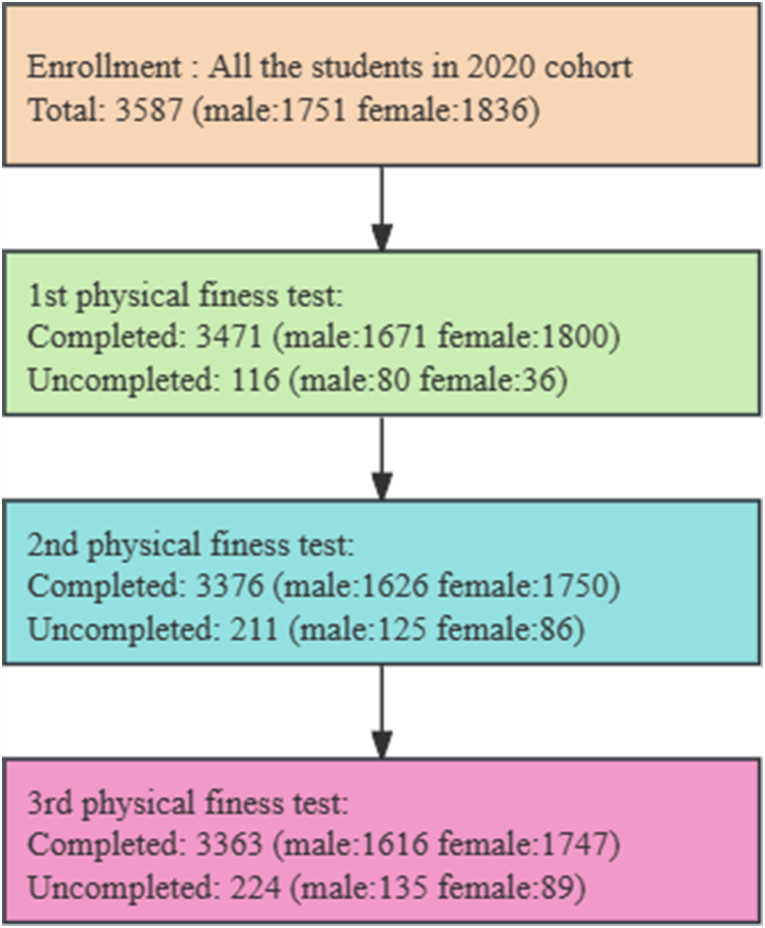
Comprehensive information on selected participants for the three physical fitness tests.

At Wannan Medical College, students are required to complete physical education courses during two academic years (four semesters) and three physical fitness tests in the first year (first semester) and the second year (first and second semesters). To ensure the safety and accuracy of the physical fitness tests, we adhered to the following criteria for student exclusion: (1) students who applied for exemption due to physical disabilities or congenital diseases(Total:148, male:75, female:73); (2) students who had to withdraw due to military enlistment(Total:64, male:53, female:11); (3) students who dropped out for other reasons(Total:12, male:7 female:5). Comprehensive information on the three physical fitness tests of the selected participants is shown in Fig. 2.

#### 2.2.2 Physical Education Teaching at Wannan Medical College

According to the requirements issued by the Chinese Ministry of Education, schools should conduct online or offline teaching based on the local COVID-19 situation. Wannan Medical College has implemented three different modes of physical education during various stages of the COVID-19 pandemic, namely, classroom teaching, online teaching, and blended teaching, in adherence to these guidelines. All the teaching modes were carried out once a week for 90 min, for 16 weeks in total. The process and description of the different physical education teaching modes are shown in Table 2.

**Table 2:** The process and description of different physical education teaching modes.

Process	Participant	Description of teaching activities		
		T1:	T2:	T3:
		Classroom Teaching	Online Teaching	Blended Teaching
		Sep 2020-Jan 2021	Sep 2021-Jan 2022	Feb 2022-Jul 2022
Before class	Teacher		Upload teaching micro video	Upload teaching micro video
	Student	Preview based on the textbook		Preview based on the textbook
			Download and study the teaching micro video	Download and study the teaching micro video
During class	Teacher	Review the previous section and teach the new chapter		Review the previous section and teach the new chapter
	Student	Learn and practice new sport skills	Sign-in through the internet learning platform	Learn and practice new sport skills
	Teacher and student	Communicate and discuss face to face	Communicate and discuss based on online learning platforms	Communicate and discuss face to face
After class	Teacher	Arrange after-school physical exercise homework	Arrange after-school physical exercise homework	Arrange after-school physical exercise homework
			Check physical exercise homework through internet learning platforms	
	Student	Complete after-school physical exercise homework	Complete after-school physical exercise homework	Complete after-school physical exercise homework
			Upload exercise videos or photos through the internet learning platforms	

##### 2.2.2.1 Stage 1: from September 2020 to January 2021

At this stage, the primary teaching mode adopted by schools is classroom teaching. For students, classroom teaching required them to preview the textbook before class, engage in physical exercise and learn sport skills during class, and complete the physical exercise tasks after class. Teachers were required to teach new chapters, guide students in physical exercise during class, and assign extracurricular physical exercise tasks after class, although there was no mandatory check of students' exercise tasks. In our study, classroom teaching covers various sports such as soccer, basketball, volleyball, badminton, table tennis, aerobics, martial arts, and yoga. Each class lasts for 90 min, consisting of 15 min of warm-up activities, 60 min of sports skill learning and practice, and 15 min of stretching and relaxation.

##### 2.2.2.2 Stage 2: from September 2021 to January 2022

At this stage, the primary teaching mode adopted by schools is online teaching. Teachers were required to upload teaching micro videos before class. During class, both teachers and students engaged in communication and discussion through the online teaching platform. Unlike in traditional teaching, teachers were tasked with assigning extracurricular physical exercise tasks, checking students' completion of these tasks after class, and providing feedback and grades. Students were expected to download teaching micro videos before class, sign in through the online teaching platform during class, complete physical exercise tasks and upload exercise videos or photos through the online teaching platform after class. The content of online physical education teaching mainly focuses on fitness guidance, aerobics, yoga, and sports appreciation. The online teaching platform included Rain Classroom, the Superstar Learning platform, Tencent QQ and WeChat. Each class lasted for 90 min, with 45 min dedicated to online learning and 45 min dedicated to offline independent practice. The 45 min of online learning comprised 15 min of micro course study and 30 min of teacher‒student Q&A.

##### 2.2.2.3 Stage 3: from February 2022 to July 2022

At this stage, the primary teaching mode adopted by schools is blended teaching. Teachers were required to upload teaching micro videos before class, cover new chapters, guide students in physical exercises during class, and perform extracurricular physical exercise tasks after class. Students were expected to download teaching micro videos and preview based on the textbook before class, and engage in physical exercises, learn sports skills during class, and complete assigned physical exercise tasks after class. However, students are no longer required to upload exercise videos or photos. The blended teaching platform includes not only the micro course presented in online teaching, but also practical aspects gained during classroom instruction. Each class lasts for 90 min, consisting of 15 min of warm-up activities, 60 min of sports skill learning and practice, and 15 min of stretching and relaxation.

### 2.3 Measurement of physical fitness

The physical fitness test was based on the "National Student physical Health Standard" of China and included 7 indicators [29], namely, body mass index, vital capacity, 50-m sprint, standing long jump, sit-and-reach, middle and long-distance running (1000-m run for male and 800-m run for female), and pull-ups for male or sit-ups for female. All physical fitness tests were conducted by the same group of teachers at Wannan Medical College. As shown in Fig. 1, each physical fitness test was administered at the end of the semester. The results of physical fitness tests were obtained from the Physical Health Center of Wannan Medical College.

#### 2.3.1 Body mass index (BMI)

Body mass index (BMI) is a widely used standard for assessing an individual's level of body fat and thinness and serves as an indicator of overall health. It is computed by dividing weight (in kilograms) by the square of height (in metres). During the measurement process, the students were instructed to remove their shoes and maintain their regular clothing for accurate recording of height and weight.

#### 2.3.2 Vital capacity

Vital capacity is a crucial indicator for assessing cardiopulmonary function. It represents the volume of air exhaled after a maximal inhalation and is measured in millilitres. Each student was recorded twice, and the best performance was taken as the student's vital capacity performance.

#### 2.3.3 50-M sprint

The 50-m run is an important index of speed and reaction ability. The data are recorded in seconds according to the amount of time an individual spends running 50 single metres. In this study, we used the speed of the 50-m sprint (m/s) to convey the outcomes. All the students initiated the sprint from a standing position, and each student's performance was recorded in a single attempt.

#### 2.3.4 Standing long jump

The standing long jump is an important index used to measure explosive power and jumping ability. It is recorded in centimetres by the farthest distance that an individual can jump in a straight line while standing. Each student underwent two measurements, and the best result was considered the student's standing long jump performance.

#### 2.3.5 Sit-and-reach

Sit-and-reach test is a crucial measure for evaluating the flexibility of the human body. It records the farthest distance, in centimetres, that an individual can reach with his or her fingertips. During the test, the students were instructed to sit on a yoga mat with their legs together, straight, toes up, and hands stretched forward.

#### 2.3.6 Middle and long-distance running

Middle and long-distance running is an important index for evaluating muscular endurance. The physical fitness test results of Chinese college students are typically represented by the 1000-m run (for males) or 800-m run (for females). The data are recorded in seconds according to the time required for an individual to run 1000 or 800 single metres. In this study, we used the speeds of middle and long-distance running (m/s) to convey the outcomes of middle and long-distance running, respectively. The testing for males and females was conducted separately on a 400-m track.

#### 2.3.7 Pull-ups (male)

Pull-up indices are important for evaluating upper body and back muscle strength in males. The number of pull-ups completed by an individual within 1 min was recorded. Students were required to face a horizontal bar, grip the bar with the palms forward, and maintain a hanging posture with straight arms. The motion involved pulling the lower jaw over the horizontal bar by raising the arms, with the student returning to the straight-arm hanging position after each pull-up.

#### 2.3.8 Sit-ups (female)

The number of sit-ups is an important index for assessing the abdominal strength of female students. The number of sit-ups completed by an individual within 1 min was recorded. During the test, the students were instructed to lie on their backs, keep their legs together, bend their legs, hold their heads with their hands, and use their abdominal muscles to contract. The task involved quickly rising to a sitting position, ensuring that their elbows touched their knees during each sit-up.

### 2.4 Statistical methods

The data were analysed with SPSS version 27.0 (IBM, Inc., Chicago, IL, USA). Descriptive statistics are expressed as the means and standard deviations or percentages. To assess the normality of the distribution of the indicator data, the Shapiro‒Wilk test was used. All the indicators followed a normal distribution. A mixed-design ANOVA model was used to evaluate the results for each physical fitness indicator. For the six identical physical fitness indicators (BMI, vital capacity, 50-m sprint, standing long jump, sit-and-reach, middle and long-distance running), analysis was conducted according to 2 (gender: male and female) × 3 (time: T1, T2, and T3). For the two different indicators (pull-ups for males or sit-ups for females), only the effect of time was examined. The Greenhouse–Geisser correction was applied when Mauchly's hypothesis test did not meet the assumption of sphericity. The main and interaction effects were analysed by post hoc tests and multiple comparisons of t tests with Bonferroni corrections. Effect size was expressed as a partial eta squared (η^2^) to determine the magnitude of the effect when significant main and interaction effects were observed. The effect magnitude was categorized as follows: 0.01 (small), 0.06 (moderate), and >0.14 (large). An alpha level of 0.05 (2-tailed) was considered to indicate statistical significance.

## 3 Results

**Fig. 3:**
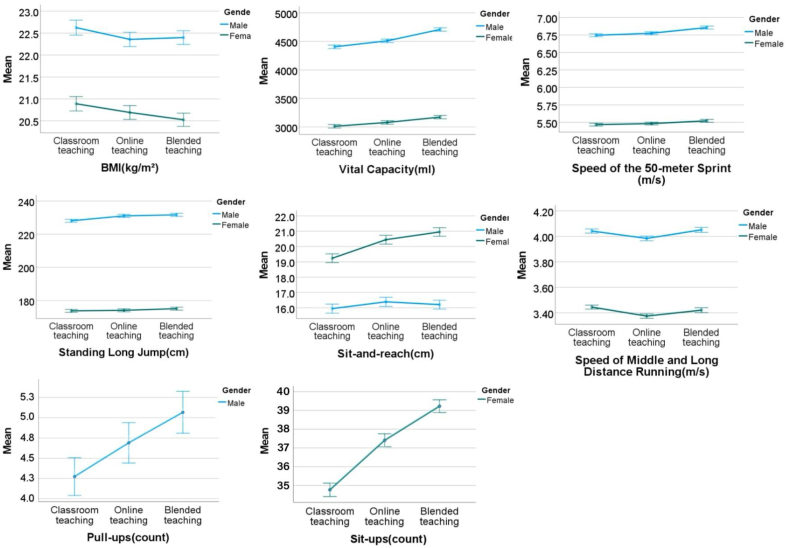
The average and 95% CI of items of the physical fitness tests for male and female participants under three different teaching modes.

A total of 3363 college students completed the physical fitness test in our study. We performed multiple comparisons of the physical fitness test results under the three teaching modes. Table 3 show the mean and standard deviation of each indicator under three different teaching modes. Fig. 3 show the average and 95% CI of the physical fitness test.

**Table 3:** Mean and standard deviation of physical fitness indicators for male and female students.

Indicators	Total(n = 3363)			Male(n = 1616)			Female(n = 1747)		
	T1	T2	T3	T1	T2	T3	T1	T2	T3
	Mean (SD)	Mean (SD)	Mean (SD)	Mean (SD)	Mean (SD)	Mean (SD)	Mean (SD)	Mean (SD)	Mean (SD)
BMI (kg/m^2^)	21.8(3.6)	21.5(3.5)	21.4(3.3)	22.6 (4.0)	22.4 (3.8)	22.4 (3.7)	20.9 (3.0)	20.7 (2.9)	20.5 (2.7)
Vital capacity(ml)	3681(954)	3767(962)	3911(984)	4404 (767)	4509 (746)	4709 (719)	3012 (527)	3080(532)	3173(501)
50-m sprint (m/s)	6.08(0.77)	6.10(0.77)	6.16(0.80)	6.75 (0.47)	6.78 (0.46)	6.86 (0.48)	5.47 (0.39)	5.49(0.77)	5.52(0.41)
Standing long jump(cm)	199.9(33.0)	201.5(34.2)	202.3(34.2)	228.1(20.8)	231.0(21.0)	231.7(20.6)	173.8(16.6)	174.1(17.1)	175.1(17.8)
Sit-and-reach (cm)	17.7(6.3)	18.5(6.5)	18.7(6.4)	15.9 (6.4)	16.4 (6.5)	16.2 (6.3)	19. 2 (5.7)	20.4(5.9)	21.0(5.6)
Middle and long distance running(m/s)	3.73(0.45)	3.67(0.48)	3.72(0.52)	4.04 (0.39)	3.98(0.44)	4.05(0.48)	3.44 (0.27)	3.37(0.3)	3.42(0.33)
pull-ups(count)				4.3(4.8)	4.7 (5.1)	5.1 (5.3)			
Sit-ups(count)							34.8 (7.6)	37.4 (7.4)	39.2 (7.2)

We examined the main effect of time to verify H_1_. For vital capacity, *F* (1.97, 6619.65) = 279.2, p < 0.001, η^2^ = 0.077. Post hoc multiple comparison analysis revealed that T1, T2, and T3 were significantly different (p < 0.001). The average vital capacity increased from 3681 mL at T1 to 3767 mL at T2 and further increased to 3911 mL at T3, showing an increasing trend. For the sit-and-reach, *F* (1.99, 6696.24) = 93.61, p < 0.001, η^2^ = 0.027. Post hoc multiple comparison analysis revealed that T1 was significantly different from T2 and T3 (p < 0.001), but there was no significant difference between T2 and T3 (p = 0.096). The average depth in the sit-and-reach increased from 17.7 cm at T1 to 18.5 cm at T2 and then increased to 18.7 cm at T3, showing an increasing trend. For middle and long-distance running, *F* (1.90, 6398.91) = 106.29, p < 0.001, η^2^ = 0.031. Post hoc multiple comparison analysis revealed that T2 was significantly different from T1 and T3 (p < 0.001), but there was no significant difference between T1 and T3 (p = 0.511). The average middle and long-distance running speeds decreased from 3.73 m/s at T1 to 3.67 m/s at T2 and then increased to 3.72 at T3, showing a trend of initial decrease and subsequent increase. For the sit-ups of females, F (1.91, 3339.98) = 415.92, p < 0.001, η^2^ = 0.192. Post hoc multiple comparison analysis revealed that T1, T2, and T3 were significantly different (p < 0.001). The average number of sit-ups increased from 34.8 at T1 to 37.4 at T2 and increased to 39.2 at T3, reflecting an increasing trend. For the pull-ups of males, *F* (1.85, 2994.79) = 32.69, p < 0.001, η^2^ = 0.020. Post hoc multiple comparison analysis revealed that T1, T2, and T3 were significantly different (p < 0.001). The average number of pull-ups increased from 4.3 at T1 to 4.7 at T2 and increased to 5.1 at T3, showing an increasing trend.

We examined the main effect of gender to verify H_2_. For BMI, *F* (1, 3361) = 253.75, p < 0.001, η^2^ = 0.070. For vital capacity, *F* (1, 3361) = 5981.08, p < 0.001, η^2^ = 0.640. For the 50-m sprint, *F* (1, 3361) = 9934.82, p < 0.001, η^2^ = 0.747. For the standing long jump, *F* (1, 3361) = 8686.73, p < 0.001, η^2^ = 0.721. For the sit-and-reach, *F* (1, 3361) = 453.91, p < 0.001, η^2^ = 0.119. For middle and long-distance running, *F* (1, 3361) = 2772.19, p < 0.001, η^2^ = 0.452. Post hoc analyses comparing the differences according to sex revealed that BMI and vital capacity were greater in males than in females (p < 0.001); the speed of 50-m sprint and middle and long-distance running was greater in males than in females (p < 0.001); and the standing long jump was greater in males than in females (p < 0.001); however, the sit-and-reach distance was greater in females than in males (p < 0.001).

We examined the main effect of time × gender to verify H_3_. For sit-and-reach, *F* (1.99, 6696.24) = 43.98, p < 0.001; η^2^ = 0.130). The average depth of the sit-and-reach for males increased from 15.9 cm at T1 to 16.4 cm at T2 and then decreased to 16.2 cm at T3, showing a trend of initial decrease and subsequent increase. Conversely, the average depth of the sit-and-reach for females increased from 19.2 cm at T1 to 20.4 cm at T2 to 21.0 cm at T3, showing an increasing trend.

## 4 Discussion

In our study, we conducted a longitudinal retrospective survey on physical education teaching modes during the COVID-19 pandemic in the 2020 cohort. The aim of this study was to assess the effects of three teaching modes on the effectiveness of students' physical fitness training. The results showed that the blended teaching demonstrated significant benefits in improving students' vital capacity, sit-and-reach, and middle and long-distance running. In addition, for gender-specific physical fitness indicators, the blended teaching also achieved significant results in female sit-ups and male pull-ups training. Notably, the online teaching was relatively ineffective in middle and long-distance running.

Our results revealed that compared to classroom teaching and online teaching, blended teaching can effectively improve most physical indicators of college students to varying degrees, especially vital capacity, sit-and-reach, and middle and long-distance running for both males and females, and sit-ups for females, and pull-ups for males. Consistent with these outcomes [30,31], blended teaching has high levels of student achievement and is more effective than purely face-to-face or purely online classes. Studies have indicated that blended teaching is more suitable for physical education, mainly for the learning of sports skills and practice [32]. In our study design, before implementing the blended teaching class, we provided students with micro videos of online teaching, which provided them with both intuitive movement demonstration learning and repeated practice guidance.

Our results also indicated that online teaching can also maintain or improve most fitness indicators. This finding is not consistent with previous studies [[33], [34], [35]]. Previous studies have shown that the physical fitness of college students has declined overall during the COVID-19 pandemic, but we found online teaching seems to maintain effect on most physical fitness indicators. Our analysis suggested that although online teaching restricts students' physical activities, but our study provided students with rich instructional content, including information about aerobic exercises, yoga, strength training, etc., and these activities have low space requirements. Furthermore, the supervision of students' physical exercise assignments also contributes to monitoring their physical activity. These factors may be the primary reasons why online teaching can maintain or improve most fitness indicators. These findings highlight the importance of online teaching during the COVID-19 pandemic and provide a valuable reference for strategy development for teaching physical education during possible future public health emergencies.

Our results also showed that the performance of middle and long-distance running under online teaching is worse compared to classroom teaching and blended teaching. Consistent with the findings of previous studies [[36], [37], [38], [39]], online teaching increased individuals sedentary and reduced daily physical exercise during the COVID-19 pandemic, resulting in a decline in students’ muscular endurance. In our study, the decline in aerobic endurance is primarily manifested in the decrease in the 1000-m run and females in the 800-m run. We believe that the decline in students' muscle endurance is not solely attributable to online physical education but rather a consequence of their excessive participation in online courses. However, under the isolating conditions of the COVID-19 pandemic, physical fitness indicators such as pull-ups, sit-ups and sit-and-reach are easier for one to perform, and as a result, these indicators were improved. However, for the 1000-m run and 800-m run, it is difficult for one to perform in limited space, which may be the primary reason for their decline, which may be the primary reason for their decline. The improvements in the 1000-m run and 800-m run after the resumption of classroom teaching further confirm this point. Therefore, we recommend that online teaching be conducted in the form of micro lessons rather than through prolonged online teaching. Furthermore, it is advisable to incorporate corresponding aerobic endurance training content into online teaching.

Our results also showed that under the same teaching mode, males generally outperformed females on most physical fitness indicators, except for sit-and-reach. This may be related to physiological differences between the sexes. In addition, blended teaching appeared to be particularly effective in improving sit-and-reach for females. Survey data from the blended teaching showed that males tended to prefer exercise classes such as basketball and soccer, while yoga and aerobics are more popular among females. This gender preference could explain why males did not see significant improvements in sit-and-reach in the blended learning. These findings emphasize the importance of considering gender differences when designing physical education classes and implementing diverse physical training strategies to meet the needs of gender-diverse students.

To our knowledge, this is the first study on the impact of three different physical education teaching modes on the physical fitness indicators of Chinese college students during the COVID-19 pandemic. Our study summarizes the physical education teaching experiences at Wannan Medical College during the COVID-19 pandemic, and the findings are crucial for refining the theoretical framework of physical education and guiding practical teaching practices.

However, our study has several limitations. First, our study was a retrospective survey on the effectiveness of college physical education, and we were unable to achieve random grouping of participants, which may introduce certain limitations to our results. Second, our study was a longitudinal survey, involving three semesters, and participant attrition as well as age factors may have an impact on physical fitness. Finally, our study sample was drawn from the same school in Anhui Province, and when considering external validity, the scope of our research needs to be expanded to ensure the generalizability of the research conclusions. In the future, it is necessary to establish effective control groups to examine the impact of different teaching modes on the physical fitness of college students. Additionally, expanding the sample size is crucial to ensure the accuracy of the research conclusions.

## 5 Conclusion

This study focused on exploring the effect of three different physical education teaching modes on the physical fitness of college students by comparing the results of physical fitness tests during the COVID-19 pandemic. The results indicate that blended teaching can effectively improve most physical indicators of college students to varying degrees and reverse the decline in middle and long-distance running performance caused by online teaching. Online teaching is particularly crucial for physical exercise not constrained by spatial limitations, such as aerobic and yoga, as these activities can effectively enhance vital capacity and sit-and-reach, pull-ups for males, and sit-ups for females. These findings emphasize the importance of conducting online physical education during unforeseen public health events and highlight the comprehensive effects of blended physical education in the post-pandemic era. Moreover, the findings emphasize the need for targeted intervention measures to address the observed variations in the levels of various physical health indicators under different physical education teaching modes.

### Ethical approval consent

Before the commencement of the study, all participants had obtained informed consent for their involvement in this research. The study was approved by Research Ethics Review Committee of Wannan Medical College (Reference Number: 2022-093), 241000, Wuhu, China.

## Funding

This research was supported by the Youth Fund for Humanities and Social Sciences Research, Ministry of Education (Grant Number: 21YJC890052). Teaching Reform Project of Anhui Provincial Department of Education (Grant Number: 2021jyxm1604). Scientific Research Project of 10.13039/501100014980Wannan Medical College (Grant Number: WKS2022Z07). Teaching Reform Project of 10.13039/501100014980Wannan Medical College (Grant Number: 2021jyxm20; 2022jbgs01).

## Data availability statement

The authors declare that the data supporting the findings of this study are available within the paper, a request for more detailed data should be sent to the corresponding authors with the permission of all authors.

## Additional information

No additional information is available for this paper.

## CRediT authorship contribution statement

**Zhixuan Tao:** Writing – review & editing, Writing – original draft, Resources, Methodology, Funding acquisition, Formal analysis, Data curation. **Ergang Zhu:** Writing – review & editing, Funding acquisition. **Xugui Sun:** Methodology, Investigation. **Jun Sun:** Writing – review & editing, Writing – original draft, Funding acquisition, Conceptualization.

## Declaration of competing interest

The authors declare that they have no known competing financial interests or personal relationships that could have appeared to influence the work reported in this paper.

## References

[1] Ke S.U.N., Changdian H.U.A.N., Huitao R.E.N., Shizhan Y.A.N., Chenglong J.I., Zhen Z.H.A.N.G., Yongshun W.A.N.G. (2020). Crisis and response: Chinese sports narrative under COVID-19 pandemic. Journal of Shanghai University of Sport.

[2] Mao S., Guo L., Li P. (2023). New era of medical education: asynchronous and synchronous online teaching during and after COVID-19. Adv. Physiol. Educ..

[3] Theoret C., Ming X. (2020). Our education, our concerns: the impact on medical student education of COVID‐19. Med. Educ..

[4] Lucey C.R., Johnston S.C. (2020). The transformational effects of COVID-19 on medical education. JAMA.

[5] Zhao L., Ao Y., Wang Y., Wang T. (2022). Impact of home-based learning experience during COVID-19 on future intentions to study online: a Chinese university perspective. Front. Psychol..

[6] Fincham D. (2013). Introducing online learning in higher education: an evaluation. Creativ. Educ..

[7] Gallè F., Sabella E.A., Ferracuti S., De Giglio O., Caggiano G., Protano C., Napoli C. (2020). Sedentary behaviors and physical activity of Italian undergraduate students during lockdown at the time of COVID− 19 pandemic. Int. J. Environ. Res. Publ. Health.

[8] Chtourou H., Trabelsi K., H'mida C., Boukhris O., Glenn J.M., Brach M., Bragazzi N.L. (2020). Staying physically active during the quarantine and self-isolation period for controlling and mitigating the COVID-19 pandemic: a systematic overview of the literature. Front. Psychol..

[9] Lavie C.J., Ozemek C., Carbone S., Katzmarzyk P.T., Blair S.N. (2019). Sedentary behavior, exercise, and cardiovascular health. Circ. Res..

[10] Lurati A.R. (2018). Health issues and injury risks associated with prolonged sitting and sedentary lifestyles. Workplace Health & Saf..

[11] Wilson O.W., Holland K.E., Elliott L.D., Duffey M., Bopp M. (2021). The impact of the COVID-19 pandemic on US college students' physical activity and mental health. J. Phys. Activ. Health.

[12] Savage M.J., James R., Magistro D., Donaldson J., Healy L.C., Nevill M., Hennis P.J. (2020). Mental health and movement behaviour during the COVID-19 pandemic in UK university students: prospective cohort study. Mental Health and Physical Activity.

[13] Filiz B., Konukman F. (2020). Teaching strategies for physical education during the COVID-19 pandemic: editor: ferman konukman. J. Phys. Educ. Recreat. Dance.

[14] Smith G.G., Ferguson D., Caris M. (2002). Teaching on-line versus face-to-face. J. Educ. Technol. Syst..

[15] Biel R., Brame C.J. (2016). Traditional versus online biology courses: connecting course design and student learning in an online setting. J. Microbiol. Biol. Educ..

[16] Kizilcec R.F., Reich J., Yeomans M., Dann C., Brunskill E., Lopez G., Tingley D. (2020). Scaling up behavioral science interventions in online education. Proc. Natl. Acad. Sci. USA.

[17] Sanders D.A., Mukhari S.S. (2023). The perceptions of lecturers about blended learning at a particular higher institution in South Africa. Educ. Inf. Technol..

[18] Van Doorn J.R., Van Doorn J.D. (2014). The quest for knowledge transfer efficacy: blended teaching, online and in-class, with consideration of learning typologies for non-traditional and traditional students. Front. Psychol..

[19] Hurlbut A.R. (2018). Online vs. traditional learning in teacher education: a comparison of student progress. Am. J. Dist. Educ..

[20] Garn A.C., Cothran D.J. (2006). The fun factor in physical education. J. Teach. Phys. Educ..

[21] Lopata C., Wallace N.V., Finn K.V. (2005). Comparison of academic achievement between Montessori and traditional education programs. J. Res. Child. Educ..

[22] Yu J., Jee Y. (2020). Analysis of online classes in physical education during the COVID-19 pandemic. Educ. Sci..

[23] American College of Sports Medicine (2024). https://www.acsm.org/education-resources/trending-topics-resources/acsm-fitness-trends.

[24] Beauchamp M.R., Hulteen R.M., Ruissen G.R. (2021). Online-delivered group and personal exercise programs to support low active older adults' mental health during the COVID-19 pandemic: randomized controlled trial. J. Med. Internet Res..

[25] Vandoni M. (2022). Effects of an online supervised exercise training in children with obesity during the COVID-19 pandemic. Int. J. Environ. Res. Publ. Health.

[26] Füzéki E., Schröder J., Groneberg D.A. (2021). Online exercise classes during the COVID-19 related lockdown in Germany: use and attitudes. Sustainability.

[27] Kabasakal E., Emiroğlu O.N. (2021). The effect of rational‐emotive education on irrational thinking, subjective wellbeing and self‐efficacy of typically developing students and social acceptance of disabled students. Child Care Health Dev..

[28] Bernard R.M., Abrami P.C., Lou Y., Borokhovski E., Wade A., Wozney L., Huang B. (2004). How does distance education compare with classroom instruction? A meta-analysis of the empirical literature. Rev. Educ. Res..

[29] National Health Commission of the People’s Republic of China (2014). Circular on the issuance of the National Physical Fitness Standards for Students.

[30] Ashraf M.A., Mollah S., Perveen S. (2022). Pedagogical applications, prospects, and challenges of blended learning in Chinese higher education: a systematic review. Front. Psychol..

[31] Bao L., Yu P. (2021). Evaluation method of online and offline hybrid teaching quality of physical education based on mobile edge computing. Mobile Network. Appl..

[32] Gubbiyappa K.S., Barua A., Das B., Murthy C.V., Baloch H.Z. (2016). Effectiveness of flipped classroom with Poll Everywhere as a teaching-learning method for pharmacy students. Indian J. Pharmacol..

[33] Ripley-Gonzalez J.W., Zhou N., Zeng T., You B., Zhang W., Liu J., Liu S. (2023). The long-term impact of the COVID-19 pandemic on physical fitness in young adults: a historical control study. Sci. Rep..

[34] Kowalsky R.J., Farney T.M., Kline C.E., Hinojosa J.N., Creasy S.A. (2023). The impact of the covid-19 pandemic on lifestyle behaviors in US college students. J. Am. Coll. Health.

[35] Mehraeen E., Karimi A., Mirghaderi P., Mirzapour P., Pashaei Z., Qaderi K., Voltarelli F. (2023). The impact of COVID-19 pandemic on the levels of physical activity: a systematic review. Infect. Disord. - Drug Targets.

[36] Zhang D., Qin C., Zhang H., Zeng X. (2020). Implementation and thoughts of online teaching of physical education in colleges and Universities under COVID-19 Epidemic. Journal of Shenyang Sport University.

[37] Pinho C.S., Caria A.C.I., Aras Júnior R., Pitanga F.J.G. (2020). The effects of the COVID-19 pandemic on levels of physical fitness. Rev. Assoc. Méd. Bras..

[38] Hallal P.C., Andersen L.B., Bull F.C., Guthold R., Haskell W., Ekelund U. (2012). Global physical activity levels: surveillance progress, pitfalls, and prospects. Lancet.

[39] Sun J., Chang J., Zhu E., Sun X., Tao Y., Chen X. (2023). Comparative research on the development of college students' physical fitness based on online physical education during the COVID-19 pandemic. BMC Publ. Health.

